# In Vitro Antimicrobial Activity of Essential Oils against *Salmonella enterica* Serotypes Enteritidis and Typhimurium Strains Isolated from Poultry

**DOI:** 10.3390/molecules24050900

**Published:** 2019-03-04

**Authors:** Valentina Virginia Ebani, Simona Nardoni, Fabrizio Bertelloni, Giovanni Tosi, Paola Massi, Luisa Pistelli, Francesca Mancianti

**Affiliations:** 1 Department of Veterinary Science, University of Pisa, Viale delle Piagge 2, 56124 Pisa, Italy; simona.nardoni@unipi.it (S.N.); fabrizio.bertelloni@unipi.it (F.B.); francesca.mancianti@unipi.it (F.M.); 2 Interdepartmental Research Center “Nutraceuticals and Food for Health”, University of Pisa, via del Borghetto 80, 56124 Pisa, Italy; luisa.pistelli@unipi.it; 3 Lombardy and Emilia Romagna Experimental Zootechnic Institute (IZSLER), Diagnostic Section of Forlì, Via Don E. Servadei 3E/3F–47122 Forlì, Italy; giovanni.tosi@izsler.it (G.T.); paola.massi@izsler.it (P.M.); 4 Department of Pharmacy, University of Pisa, via Bonanno 6, 56126 Pisa, Italy

**Keywords:** *Salmonella* Enteritidis, *Salmonella* Typhimurium, *Saccharomyces cerevisiae*, poultry, essential oils, antimicrobial activity

## Abstract

*Salmonella enterica* serotype Enteritidis and *S. enterica* serotype Typhimurium are frequently present among poultry and are associated with outbreaks of human salmonellosis. The study investigated the in vitro antimicrobial activity of essential oils (EOs) obtained from *Aloysia triphylla*, *Cinnamomum zeylanicum*, *Cymbopogon citratus*, *Litsea cubeba*, *Mentha piperita*, *Syzygium aromaticum* against *S.* Enteritidis and *S.* Thyphimurium strains previously isolated from poultry. A 1:1 mixture of *C. zeylanicum* and *S. aromaticum* was also tested. The activity of all compounds was evaluated against the yeast *Saccharomyces cerevisiae*, commonly used as probiotic. The highest antibacterial activity was observed for *C. zeylanicum* (minimum inhibitory concentrations (MICs) ranging from 1.26 mg/mL to 0.63 mg/mL), *S. aromaticum* (MICs from 2.637 mg/mL to 0.164 mg/mL) and the mixture (MICs from 1.289 mg/mL to 0.322 mg/mL). No activity was recorded against *S. cerevisiae.* The results suggest a possible use of *C. zeylanicum* and *S. aromaticum*, alone or in combination, in the farm environment for disinfection and in poultry diet, combined with *S. cerevisiae* administration, for an integrated approach to avoid *Salmonella* intestinal colonization.

## 1. Introduction

Genus *Salmonella*, belonging to the family *Enterobacteriaceae*, includes the species *S. enterica* and *S. bongori* in which several serotypes have been classified [1].

Pullorum disease and fowl typhoid are avian host-specific salmonellosis due to *S. enterica* serotype Pullorum and *S. enterica* serotype Gallinarum, respectively.

All the other serotypes may cause avian paratyphoid. Among them, *S. enterica* serotype Enteritidis (*S*. Enteritidis) and *S. enterica* serotype Typhimurium (*S*. Typhimurium) are the most widespread among poultry and often associated to outbreaks of human salmonellosis [2].

Chickens infected by paratyphoid salmonellae may be depressed, reluctant to move, and exhibit symptoms of diarrhea; with decreased egg production sometimes observed in laying hens. Chickens can harbor paratyphoid salmonellae without showing clinical signs and disseminate the pathogen in the environment [3]. Moreover, salmonellae intestinal colonization of poultry causes egg contamination, and carcass contamination during slaughter. For these reasons, humans may contract the infection mainly through the consumption of contaminated eggs and poultry meat [4].

Probiotics yeasts, such as *Saccharomyces* sp. are known to protect the intestinal tract of hosts [5,6] and to modulate the immune response against pathogens such as *S.* Typhimurium [7].

Essential oils (EOs) have been shown to have antibacterial and antifungal activity [8]. Moreover, it has been demonstrated that EOs have a positive effect on the production performance of broiler chickens which is reflected in reduced feed intake, increased body weight gains, greater immunity, and better health [9,10].

The aim of the present study was to evaluate the antimicrobial activity of EOs obtained from lemon verbena (*Aloysia triphylla* L’Hèr. Britton), cinnamon (*Cinnamomum zeylanicum* J. Presl), lemongrass (*Cymbopogon citratus* (DC.) Stapf), litsea (*Litsea cubeba* (Lour.) Pers.), peppermint (*Mentha piperita*) and clove (*Syzygium aromaticum* (L.) Merr. and L.M. Perry) and a 1:1 mixture composed by *C. zeylanicum* and *S. aromaticum* against *S.* Enteritidis and *S.* Thyphimurium strains, previously isolated from poultry. Moreover, their activity against *Saccharomyces cerevisiae* was checked to evaluate the impact of these natural products (alone or in combination) on this probiotic yeast, to yield an integrated control of *Salmonella* infections in poultry breeding. These EOs and mixture were selected on the basis of their biological activity on bacterial and fungal agents characterized by a huge impact on poultry breeding [8].

At the best of our knowledge, this is the first study evaluating the action of these EOs with an inclusion of a mixture versus several *S.* Enteritidis and *S.* Typhimurium strains isolated from poultry.

## 2. Results

### 2.1. Essential Oil Composition

Table 1 shows the composition of the six analyzed EOs and the assembled mixture. GC-MS analysis detected several compounds for each tested EO. In detail, 17 main compounds were identified in *M. piperita* and *A. triphylla*, 15 in *C. zeylanicum*, 14 in *L. cubeba*, 11 in *C. citratus*, and 4 in *S. aromaticum.* Dominant compounds were mostly monoterpenes. Menthone, menthol and menthofuran were prevalent in *M. piperita*, limonene, sabinene and citronellal in *A. triphylla*, geranial and neral in *C. citratus*, geranial, neral and limonene in *L. cubeba.* Phenylpropanoides were the main constituents of the two EOs with the most relevant antibacterial activity: *C. zeylanicum* (cynnamaldehyde) and *S. aromaticum* (eugenol and eugenyl acetate).

### 2.2. Antimicrobial Activity

#### 2.2.1. Agar Disk Diffusion Method

Assayed EOs and the mixture showed different degrees of growth inhibition against the eighteen tested *Salmonella* isolates (Table 2). *C. zeylanicum* and *S. aromaticum*, alone or in combination, induced the largest inhibition zones versus almost all the evaluated strains, whereas low antibacterial activity was observed for the remaining EOs. The overall inhibition zone for tested EOs ranged from 7.0 mm to 17.0 mm against *S.* Enteritidis strains. The lowest potential was observed in *A. triphylla*, *C. citratus*, *L. cubeba* and *M. piperita* EOs, where inhibition zone average was 7.0 mm. *C. zeylanicum* and *S. aromaticum*, alone or in combination, induced the largest inhibition zones versus almost all the evaluated strains. The inhibition zone for *C. zeylanicum* ranged from 7.0 mm to 17.0 mm, for *S. aromaticum* from 9.0 mm to 13.0 mm and for the mixture from 10.0 mm to 17.0 mm.

*S.* Enteritidis 232 was found to be the least sensitive isolate to *C. zeylanicum* and *S. aromaticum* alone or in combination; *S.* Enteritidis 233 was found to be poorly sensitive to *C. zeylanicum* and *S. aromaticum*, but showed a large inhibition zone (15.0 mm) when tested with the mixture. The strains 234 and 236 appeared to be the most sensitive to these EOs.

The tested *S.* Typhimurium isolates were less sensitive to the assayed EOs when compared with *S.* Enteritidis strains. In fact, inhibition zones ranged from 7.0 mm to 13.0 mm. The lowest antimicrobial activity was observed in *A. triphylla*, *C. citratus*, *L. cubeba* and *M. piperita* EOs, with an average inhibition zone of 7.0 mm. *S.* Typhimurium 261 and 176 were the most sensitive strains to *C. zeylanicum* showing inhibition zones of 10.0 mm and 13.0 mm, respectively, whereas the remaining *S.* Typhimurium strains had zones of 7.0 mm. The inhibition zone ranged from 9.0 mm to 11.0 mm for *S. aromaticum* and from 9.0 mm to 13.0 mm for the mixture.

DMSO, tested as negative control, did not result in inhibition zone growth, whereas chloramphenicol, used as positive control, was found to be effective against all the isolates.

#### 2.2.2. Minimum Inhibitory Concentration

Table 3 reports the minimum inhibitory concentration (MIC) values, expressed both as percentage and as mg/mL, testing EOs and the mixture versus the *Salmonella* isolates. *C. zeylanicum*, *S. aromaticum* and their mixture showed good activity against all the selected strains, with MICs ranging from 1.26 mg/mL to 0.63 mg/mL for *C. zeylanicum*, from 2.637 mg/mL to 0.164 mg/mL for *S. aromaticum* and from 1.289 mg/mL to 0.322 mg/mL for the mixture. The remaining EOs showed a weak activity: 17.1 mg/mL for *A. triphylla*, 17.9 mg/mL for *C. citratus*, 17.7–8.85 mg/mL for *L. cubeba* and 18.24–9.12 mg/mL for *M. piperita*.

No growth inhibition was observed with the negative control, whereas chloramphenicol inhibited the growth of all strains.

*S. cerevisiae* showed an overall low sensitivity against all the tested EOs. *A. triphylla* had the lowest MIC value (5%, 8.55 mg/mL), while *S. aromaticum* was found to be completely ineffective at a 10% dilution. The undiluted mixture also did not yield any antimycotic effect.

## 3. Discussion

The results obtained in the present investigation show that *S. aromaticum* and *C. zeylanicum* EOs in combination have good antibacterial activity versus both *S.* Enteritidis and *S.* Typhimurium isolates.

Previous studies demonstrated the antibacterial effect of *S. aromaticum* and *C. zeylanicum* EOs due to the presence of several constituents. In particular, eugenol has been proven to be a component of *S. aromaticum* EO, with a large spectrum of antibacterial and antifungal effects [11]. The *S. aromaticum* EO employed in the present study had a relevant amount (77.9%) of this component, which could have determined the antibacterial effect.

*C. zeylanicum* activity has been attributed to cinnamaldehyde and eugenol, substances that react with lipid and hydroxyl radicals converting them into stable products through their hydrogen-donating ability [12]. Moreover, these components are able to inhibit the production of essential enzymes by the bacteria due to the presence of a carbonyl group that binds and inactivates them and/or causes damage to the cell wall of the bacteria [13]. EO from *C. zeylanicum* used in our investigation had 3% eugenol and 56.4% cinnamaldehyde (which represents its main compound) content. The presence of both constituents may have enhanced the antibacterial effect, as suggested by Burt et al. [14], who described a higher antimicrobial activity of *C. zeylanicum* EO compared with cinnamaldehyde alone.

EOs from *A. triphylla*, *C. citratus*, *L. cubeba* and *M. piperita* showed no relevant activity against *Salmonella*. Other authors reported in vitro antibacterial activity of *S. aromaticum* and *C. zeylanicum* EOs against paratyphoid *Salmonella* strains [15,16,17]. However, our study evaluated the action of different EOs versus several *S.* Enteritidis and *S.* Typhimurium strains isolated from poultry, whereas very scant data about the activity of EOs from *A. triphylla*, *C. citratus*, *L. cubeba* and *M. piperita* against *Salmonella* are available in literature [18,19,20].

All the EOs tested in this study had been previously assayed against an *Escherichia coli* strain: *C. zeylanicum* had an MIC value of 2.52 mg/mL, *S. aromaticum* of 1.318 mg/mL and their blend of 2.578 mg/mL [8]. The activity against the different *Salmonella* isolates resulted as being much higher, in fact MIC values decreased to 0.63 mg/mL, 0.164 mg/mL and 0.322 mg/mL, respectively.

*S. cerevisiae* is an ascomycete yeast used as a feed additive commonly sold on the market, which is reported to increase macrophage activation, as well as intestinal immune modulating activity and to have an anti-stress effect [21]. *A. triphylla* showed a limited antifungal action when compared with its antimycotic activity against *A. fumigatus* [8]. The activity of this EO would be related to the large amount of limonene, sabinene and citronellal. These compounds strongly inhibit *C. albicans* [22,23,24]. However, *S. cerevisiae* showed a reduced sensitivity versus other EOs provided of antifungal activity, such as *L. cubeba* and *C. citratus.* Our results are not in agreement with literature, when referred to a striking antimycotic activity of *C. citratus* [25], as well as *S. aromaticum* [26], and *M. piperita* [27], while data about *L. cubeba* and *C. zeylanicum* are not available.

## 4. Material and Methods

### 4.1. Essential Oils

Essential oils from the following six plants were used in this experiment: lemon verbena (*Aloysia triphylla* L’Hèr. Britton), cinnamon (*Cinnamomum zeylanicum* J. Presl), lemongrass (*Cymbopogon citratus* (DC.) Stapf), litsea (*Litsea cubeba* (Lour.) Pers.), peppermint (*Mentha piperita*), and clove (*Syzygium aromaticum* (L.) Merr. and L.M. Perry). All EOs, purchased by the producer (FLORA^®^, Pisa, Italy), were maintained at 4 °C in dark glass vials until their employment.

EOs quality control for antibacterial and antimycotic activity was tested before experiment. EOs were streaked onto a blood agar plate. The plates were incubated at 37 °C for 48 h. Absence of colonies after the incubation period confirmed the EOs sterility.

A 1:1 mixture with *C. zeylanicum* and *S. aromaticum* was prepared and employed in the study.

#### Essential Oils Analysis

All the selected EOs and the mixture were analyzed by GC-MS according to the method previously described [28]. Briefly, the analysis was performed with a Varian CP-3800 apparatus (Varian Inc., Palo Alto, CA, USA) equipped with a DB-5 capillary column (30m × 0.25 mm i.d., film thickness 0.25 μm) and a Varian Saturn 2000 ion-trap mass detector (Varian Inc.). The oven temperature was programmed rising from 60 °C to 240 °C at 3 °C/min; injector temperature 220 °C; transfer-line temperature 240 °C; carrier gas He (1 mL/min).

### 4.2. Antimicrobial Activity

#### 4.2.1. Microbial Strains

Nine *S*. Enteritidis and nine *S*. Typhimurium isolates were tested in vitro for antimicrobial sensitivity. All strains were previously isolated from poultry and kept in collection at −80 °C in glycerol broth. Each strain was inoculated into brain hearth infusion broth (BHIB, Oxoid Ltd., Basingstoke, Hampshire, UK) and incubated at 37 °C for 24 h. Cultures of 1–2 × 10^7^ CFU/mL, corresponding to 0.5 McFarland standard, were employed in the tests.

Antifungal activity of EOs and the mixture was evaluated on a yeast strain of *S. cerevisiae*, characterized by ID32C galleries (BioMerieux, Lyon, France).

#### 4.2.2. Agar Disk Diffusion Method

Antibacterial activity of the selected EOs and the mixture was tested by Kirby-Bauer agar disk diffusion method following the procedures reported by Clinical and Laboratory Standards Institute (CLSI) [29]. Briefly, EOs and the mixture were diluted 1:10 in dimethyl sulfoxide (DMSO, Oxoid Ltd.) and one absorbent paper disk was impregnated with 10 µl of each dilution, respectively, and tested against each isolate. In this way 10 µl for each disk had 171 µg for *A. triphylla*, 202 µg for *C. zeylanicum*, 179 µg for *C. citratus*, 177 µg for *L. cubeba*, 182 µg for *M. piperita*, 211 µg for *S. aromaticum*, 101 µg (*C. zeylanicum*) and 105 µg (*S. aromaticum*) for the mixture.

A paper disk impregnated with 10 µl of DMSO was included as negative control. A commercial disk impregnated with chloramphenicol (30 µg) (Oxoid Ltd.) was used as positive control. Growth inhibition zones were calculated after incubation at 37 °C for 24 h. All tests were performed in triplicate.

The in vitro sensitivity of all *Salmonella* strains to chloramphenicol (30 µg) (Oxoid Ltd.) was assayed by the same method and the results were interpreted as indicated by CLSI [30].

#### 4.2.3. Minimum Inhibitory Concentration

Minimum inhibitory concentration (MIC) was determined for all EOs and the mixture with the broth microdilution method, starting from a dilution of 10% (*v*/*v*) and following the guidelines of CLSI [31] for bacteria and CLSI M27A_3_ for yeasts [32], and a protocol previously reported [20]. The MIC value was determined as the lowest concentration, expressed in mg/mL, for each EO and the mixture at which bacteria and yeast showed no visible growth. The same assay was performed simultaneously for microorganisms growth control (tested agents and media) and sterility control (tested oil or mixture and media).

All tests were performed in triplicate and using chloramphenicol (Oxoid Ltd.) and 5-fluorocytosine (Oxoid Ltd.) as controls.

## 5. Conclusions

*C. zeylanicum* and *S. aromaticum* EOs in a 1:1 mixture seem to be promising natural products to be employed against field *S.* Enteritidis and *S.* Typhimurium strains affecting poultry. Although they cannot be used for therapeutic scope, they could be applied in environmental disinfection considering their additional activity against *E. coli*. Moreover, these EOs could be added in feed to prevent intestinal colonization in chickens. Use of these EOs in poultry diets would not interfere with *S. cerevisiae* used as a probiotic, presenting an integrated tool of prevention.

## Figures and Tables

**Table 1:** Chemical composition of the essential oils (EOs) tested, expressed as percentage.

Chemical Component	LRI	*Aloysia triphylla*	*Cymbopogon citratus*	*Cinnamomum zeylanicum*	*Litsea cubeba*	*Mentha piperita*	*Syzygium aromaticum*	Mixture
α-Thujene	930	0.2	0.1	0.3				
α-Pinene	939					0.8		
Sabinene	975	24.0		0.1	1.0	1.8		
β-Pinene	979			0.5	1.2			0.2
α-Phellandrene	1003			2.1				0.3
α-Terpinene	1017	0.2		1.0		0.2		0.1
*p*-Cymene	1025	0.4		3.0	0.2	0.4		0.5
Limonene	1029	36.7	2.0		16.3	3.0		
β-Phellandrene	1030			5.9				1.1
1,8-Cineole	1031		0.3		2.3	5.0		
γ-Terpinene	1060	0.3			0.1	0.3		
Terpinolene	1089	0.1		0.3		0.1		
Linalool	1097	3.0	1.5	6.3	1.5	0.4		1.5
Menthone	1153					26.6		
Citronellal	1153	12.0	0.5		0.9			
Menthofuran	1164					12.5		
Menthol	1172					32.4		
4-Terpineol	1177	0.7		0.3	0.1			0.1
α-Terpineol	1189	0.4		0.8	0.5	0.3		0.3
Citronellol	1226	1.9						
Neral	1238	0.7	35.2		32.5			
Geraniol	1253		4.4		0.5			
Geranial	1267	1.2	38.4		36.4			
(*E*)-Cinnamaldehyde	1270			56.4				18.5
Menthyl acetate	1295					6.1		
Eugenol	1359			3.0			77.9	51.7
β-Caryophyllene	1419	1.3	2.3	10.3	0.8	2.8	8.9	7.6
Germacrene D	1485	0.7	0.2			0.7		
Eugenyl acetate	1523						12.2	12.7
δ-Cadinene	1523		0.3	0.2		0.2	0.2	0.9
τ-Cadinol	1640	0.1						
Unknown		0.5	0.4	0.3	0.6	0.2		0.1
Total		99.5	99.6	99.7	99.4	99.8	100.0	99.9
Monoterpene Hydrocarbons (MH)		66.0	3.9	15.5	21.3	6.9		2.2
Oxygenated Monoterpenes (OM)		26.4	86.3	7.4	75.7	87.8		1.9
Sesquiterpene Hydrocarbons (SH)		4.7	4.5	14.7	0.9	4.6	9.5	10.1
Oxygenated Sesquiterpenes (OS)		1.9	0.9	0.8		0.3	0.4	1.2
Phenylpropanoides (PP)			2.0	60.3			90.1	64.4
Non-terpenes (NT)		0.5	2.0	1.0	1.5	0.2		20.1

Legend—LRI: Linear retention indices on the DB5 column; Mixture: *Cinnamomum zeylanicum* and *Syzygium aromaticum*.

**Table 2:** The growth inhibition zones (expressed in mm) obtained testing the selected *Salmonella* Enteritidis and *Salmonella* Typhimurium strains against the assayed EOs and the mixture.

Bacterial Strain	Essential Oil							Chloramphenicol
	*Aloysia triphylla*	*Cinnamomu zeylanicum*	*Cymbopogon citratus*	*Litsea cubeba*	*Mentha piperita*	*Syzygium aromaticum*	Mixture	
	M SD	M SD	M SD	M SD	M SD	M SD	M SD	
*S*. Enteritidis 217	7.0 ± 0.0	11 ± 0.0	7.0 ± 0.0	7.0 ± 0.0	7.0 ± 0.0	9.0 ± 0.0	12 ± 0.6	19 (S)
*S*. Enteritidis 218	7.0 ± 0.0	12 ± 0.6	7.0 ± 0.0	7.0 ± 0.0	7.0 ± 0.0	10 ± 0.0	12 ± 0.0	18 (S)
*S*. Enteritidis 219	7.0 ± 0.0	13 ± 1.0	7.0 ± 0.0	7.0 ± 0.0	7.0 ± 0.0	12 ± 1.0	13 ± 0.6	20 (S)
*S*. Enteritidis 220	7.0 ± 0.0	7.0 ± 0.0	7.0 ± 0.0	7.0 ± 0.0	7.0 ± 0.0	13 ± 1.0	11 ± 0.0	20 (S)
*S.* Enteritidis 221	7.0 ± 0.0	8.0 ± 0.0	7.0 ± 0.0	7.0 ± 0.0	8.0 ± 0.0	12 ± 0.6	13 ± 1.0	19 (S)
*S*. Enteritidis 232	7.0 ± 0.0	7.0 ± 0.0	7.0 ± 0.0	7.0 ± 0.0	7.0 ± 0.0	10 ± 0.6	10 ± 0.0	19 (S)
*S*. Enteritidis 233	7.0 ± 0.0	7.0 ± 0.0	7.0 ± 0.0	7.0 ± 0.0	7.0 ± 0.0	9.0 ± 0.0	15 ± 1.0	19 (S)
*S*. Enteritidis 234	7.0 ± 0.0	17 ± 0.6	7.0 ± 0.0	7.0 ± 0.0	7.0 ± 0.0	9.0 ± 0.0	17 ± 0.6	21 (S)
*S.* Enteritidis 236	7.0 ± 0.0	17 ± 0.6	7.0 ± 0.0	7.0 ± 0.0	7.0 ± 0.0	9.0 ± 0.0	16 ± 1.0	18 (S)
*S*. Typhimurium 240	7.0 ± 0.0	7.0 ± 0.0	7.0 ± 0.0	7.0 ± 0.0	7.0 ± 0.0	9.0 ± 0.0	10 ± 0.0	20 (S)
*S.* Typhimurium 241	7.0 ± 0.0	7.0 ± 0.0	7.0 ± 0.0	7.0 ± 0.0	7.0 ± 0.0	9.0 ± 0.0	11 ± 0.0	21 (S)
*S.* Typhimurium 245	7.0 ± 0.0	7.0 ± 0.0	7.0 ± 0.0	7.0 ± 0.0	7.0 ± 0.0	9.0 ± 0.0	9 ± 0.0	21 (S)
*S.* Typhimurium 250	7.0 ± 0.0	7.0 ± 0.0	7.0 ± 0.0	7.0 ± 0.0	7.0 ± 0.0	9.0 ± 0.0	10 ± 0.6	19 (S)
*S.* Typhimurium 251	7.0 ± 0.0	7.0 ± 0.0	7.0 ± 0.0	8.0 ± 0.0	8.0 ± 0.0	10 ± 0.0	10 ± 0.0	20 (S)
*S.* Typhimurium 252	7.0 ± 0.0	7.0 ± 0.0	7.0 ± 0.0	8.0 ± 0.0	7.0 ± 0.0	9.0 ± 0.0	12 ± 0.6	19 (S)
*S.* Typhimurium 258	7.0 ± 0.0	7.0 ± 0.0	7.0 ± 0.0	8.0 ± 0.0	7.0 ± 0.0	10 ± 0.6	13 ± 1.0	18 (S)
*S*. Typhimurium 261	7.0 ± 0.0	10 ± 0.0	7.0 ± 0.0	7.0 ± 0.0	8.0 ± 0.0	11 ± 0.6	11 ± 0.6	19 (S)
*S.* Typhimurium 176	7.0 ± 0.0	13 ± 0.6	7.0 ± 0.0	7.0 ± 0.0	8.0 ± 0.0	9.0 ± 0.0	13 ± 0.6	19 (S)

Legend—M: mean expressed in mm; SD: standard deviation; S: susceptible; Mixture: *Cinnamomum zeylanicum* and *Syzygium aromaticum*.

**Table 3:** Minimum inhibitory concentration (MIC) values of tested EOs and the mixture, expressed as both percentage and mg/mL, against selected *Salmonella* Enteritidis, *Salmonella* Typhimurium and *Saccharomyces cerevisiae* isolates.

Bacterial Strain	Essential Oil														Chloramphenicol
	*Aloysia triphylla*		*Cinnamomum zeylanicum*		*Cymbopogon citratus*		*Litsea cubeba*		*Mentha piperita*		*Syzygium aromaticum*		Mixture		
	%	mg/mL	%	mg/mL	%	mg/mL	%	mg/mL	%	mg/mL	%	mg/mL	%	mg/mL	µg/mL
*S*. Enteritidis 217	10	17.1	0.3	0.63	10	17.9	10	17.7	10	18.24	1.25	2.637	0.15	0.644	8
*S*. Enteritidis 218	10	17.1	0.6	1.26	10	17.9	10	17.7	10	18.24	0.3	0.659	0.15	0.644	6
*S*. Enteritidis 219	10	17.1	0.3	0.63	10	17.9	10	17.7	10	18.24	0.3	0.659	0.15	0.644	6
*S*. Enteritidis 220	10	17.1	0.3	0.63	10	17.9	10	17.7	10	18.24	0.3	0.659	0.15	0.644	7
*S.* Enteritidis 221	10	17.1	0.3	0.63	10	17.9	10	17.7	5	9.12	0.6	1.318	0.15	0.644	8
*S*. Enteritidis 232	10	17.1	0.6	1.26	10	17.9	10	17.7	10	18.24	0.6	1.318	0.15	0.644	7
*S*. Enteritidis 233	10	17.1	0.6	1.26	10	17.9	10	17.7	10	18.24	0.15	0.329	0.07	0.322	7
*S*. Enteritidis 234	10	17.1	0.6	1.26	10	17.9	10	17.7	10	18.24	0.15	0.329	0.07	0.322	7
*S.* Enteritidis 236	10	17.1	0.6	1.26	10	17.9	10	17.7	10	18.24	0.07	0.164	0.07	0.322	5
*S*. Typhimurium 240	10	17.1	0.3	0.63	10	17.9	10	17.7	10	18.24	1.25	2.637	0.3	1.289	6
*S.* Typhimurium 241	10	17.1	0.6	1.26	10	17.9	10	17.7	10	18.24	0.6	1.318	0.3	1.289	6
*S.* Typhimurium 245	10	17.1	0.3	0.63	10	17.9	10	17.7	10	18.24	0.6	1.318	0.3	1.289	8
*S.* Typhimurium 250	10	17.1	0.3	0.63	10	17.9	10	17.7	10	18.24	0.3	0.659	0.3	1.289	7
*S.* Typhimurium 251	10	17.1	0.3	0.63	10	17.9	5	8.85	5	9.12	0.07	0.164	0.3	1.289	5
*S.* Typhimurium 252	10	17.1	0.3	0.63	10	17.9	10	17.7	10	18.24	0.3	0.659	0.15	0.644	6
*S.* Typhimurium 258	10	17.1	0.3	0.63	10	17.9	5	8.85	10	18.24	0.07	0.164	0.15	0.644	6
*S*. Typhimurium 261	10	17.1	0.6	1.26	10	17.9	10	17.7	5	9.12	0.15	0.329	0.3	1.289	7
*S.* Typhimurium 176	10	17.1	0.6	1.26	10	17.9	10	17.7	10	18.24	1.25	2.637	0.3	1.289	5
*S. cerevisiae*	5	8.55	10	20.2	7.5	13.42	7.5	13.27	10	18.24	ne		ne		0.20 *

Legend—ne: not effective; *: 5-fluorocytosine; Mixture: *Cinnamomum zeylanicum* and *Syzygium aromaticum*.
